# Familial Mediterranean Fever: Diagnosing as Early as 3 Months of Age

**DOI:** 10.1155/2014/296479

**Published:** 2014-03-27

**Authors:** Gonca Keskindemirci, Nuray Aktay Ayaz, Esin Aldemir, Çiğdem Aydoğmuş, Gönül Aydoğan, Sultan Kavuncuoğlu

**Affiliations:** İstanbul Kanuni Sultan Süleyman Educational and Research Hospital, 34303 İstanbul, Turkey

## Abstract

Familial Mediterranean Fever is an autosomal recessive disease. Major symptoms of disease are recurrent fever accompanied by serositis attacks. The disease is usually diagnosed before 20 years of age. Symptoms related to FMF are noted when children become more verbal, usually after 2 years of age. In this case report, the youngest patient with the diagnosis of FMF is presented. She was consulted to pediatric rheumatology for the high acute phase response and fever. It was learned that her mother had recurrent swelling of her ankle joints. Mutation analysis was performed and two homozygous mutations (M694V and R202Q) were identified. She was diagnosed as FMF at 3 months of age and colchicine was started. She responded to colchicine. Her uncontrolled acute phase response declined gradually. This case was reported to point out the importance of early remembrance of autoinflammatory diseases even at very early ages especially at endemic countries.

## 1. Introduction

Familial Mediterranean Fever (FMF) is an autosomal recessively inherited autoinflammatory disorder characterized by recurrent attacks of fever and inflammation of serosal membranes and it is mainly affecting Jews, Armenians, Turks, Arabs, and ethnic groups living around the Mediterranean basin [1]. Febrile episodes which are accompanied by peritonitis, pleuritis, synovitis, splenomegaly, hepatomegaly, myalgia, erysipelas-like erythema, protracted febrile myalgia, and seizures are clinical features of FMF [2]. Onset of the symptoms of the disease begins usually before 20 years of age [3]. The diagnosis is based on clinical features because there are no disease specific laboratory findings. Mutation analysis helps and supports diagnostic evaluation. Symptoms related to FMF are noted when children become more verbal, usually after 2 years of age. Before this age, FMF awareness is unsatisfactory and there are only few publications about cases diagnosed before 2 years of age.

## 2. Case

A 3-month-old girl was brought to the pediatric rheumatology unit for the complaints of recurrent fever and episodes of vomiting. From her history, it was learned that she was admitted to the hospital at the 6th day of life with symptoms of vomiting and restlessness. At that time, her CRP level was high and she had leukocytosis. Sepsis work-up and lumbar puncture were performed after maternal cultures were taken. With the diagnosis of sepsis, antibiotic therapy (ampicillin-sulbactam and netilmicin) was commenced. Hemoculture and CSF cultures were negative. Her CRP levels went on rising but she was clinically silent. At the 10th day of the antibiotic therapy, due to increasing CRP levels, lumbar puncture was repeated, but again CSF culture was sterile. Her antibiotic therapy was changed to ampicillin-sulbactam and cefotaxime. Due to her consistently high acute phase reactants therapy was switched to tazobactam and amikacin. At the 10th day of this therapy, CRP level had continued to be high. Therefore, amikacin was switched to meropenem. At the 28th day of the hospitalization, lumbar puncture was repeated. There was no growth in the BOS culture. Due to high CRP levels, vancomycin was added to meropenem. Cranial and abdominal ultrasounds were normal. Echocardiography was normal too. MRSA growth was detected in the blood culture. At the 43th day of hospitalization, CRP level was 24 mg/dL and CSF culture and hemoculture were sterile. Externalization from the neonatology unit was planned but her CRP level had increased to 70 mg/dL at the 50th day. Procalcitonin levels were normal. Pediatric immunology consultation was requested. Immunoglobulin levels and lymphocyte subgroups were normal. Echocardiography and cranial and abdominal ultrasonography were normal. Urinary mevalonic acid and immunoglobulin D levels were normal. Autoantibodies were negative. At the 60th day of admission, she was externalized from the hospital according to family's will with a CRP level of 35 mg/dL. She stayed two weeks at home and she was brought to the hospital with fever, restlessness, and irritability. She had pyuria, leukocytosis, and high level of CRP. She was admitted to the pediatric infectious disease clinic with the diagnosis of urinary tract infection and ceftriaxone therapy was started. Urinary culture and hemoculture were sterile and renal ultrasound was normal. Until she was referred to pediatric rheumatology unit, she had 2 febrile episodes with vomiting and irritability. An autoinflammatory syndrome was suspected. As her serum IgD and urine mevalonic acid levels were normal and she was a Turkish child, first considered disease was FMF. A thorough history of the patient and family were obtained and it was learned that her mother was having recurrent episodes of arthralgia, myalgia, and arthritis. Her physical examination was normal. MEFV gene mutations were studied. Although she had a high CRP level, she was externalized at the 10th day of antibiotic therapy. She was found to be homozygous for both M694V and R202Q. Her mother was also tested and she had the same mutations. Colchicine therapy was started with a dose of 0.25 mg/day. At the second week of colchicine therapy, she was clinically normal and her CRP level was within normal limits. No attack was reported after colchicine treatment. Her CRP, erythrocyte sedimentation rate, and serum amyloid A levels were normal at the end of her first age.

## 3. Discussion

FMF is an autosomal recessive autoinflammatory disease with the manifestations of recurrent fever, abdominal pain, joint pain, and swelling and chest pain. Recurrent fever during early childhood may be the only manifestation of FMF [4, 5]. Abdominal attacks are reported in 90%, articular attacks in 75%, and pleural attacks in 45% of affected individuals [4]. Symptoms of the disease start before 20 years of age in 90% of patients and 60% begin before the age of 10. Onset of the disease at older ages occurs rarely [6]. There is a paucity of literature about FMF before 2 years of age, because of the vagueness of symptoms in this age period. The diagnosis is usually difficult due to the insufficiency of the verbal capabilities in infants and toddlers. So younger patients cannot express their pain verbally and they express themselves usually with restlessness and agitation. At the early ages, especially in the first year of life, fever is the most common symptom, but it is attributed to infectious diseases.

MEFV gene which is located on the short arm of chromosome 16 was first defined by two groups in 1997 [7, 8]. Presence of mutations supports the diagnosis of FMF. These mutations are important when the physicians do not feel certain about their diagnosis. In the literature, homozygosity for M694V mutation correlates with younger age of FMF onset and increased disease severity [5, 9, 10]. M694V is more commonly seen among Sephardic Jews, Turks, and Armenians [11]. Similar to these reports, Yalçinkaya et al. and Tunca also had shown that the most frequent three mutations in Turkısh patients were M694V, M680I, and V726A [12, 13]. Physicians have not been able to discharge our patient from hospital due to high acute phase response and unremitting course of her fever. It had been diagnosed as sepsis, meningitis, and urinary tract infection, respectively. But her CRP levels did not respond to multiantibiotic therapies. With a thorough history from her family, the possible diagnosis of an autoinflammatory disease was made. As she was from Turkish origin, the most sensible diagnosis would be FMF. She was also found to be homozygous for M694V and R202Q mutations. This was compatible with the literature. She quickly responded to colchicine both in clinical and laboratory basis.

As our patient, the delay in diagnosis may result with unnecessary hospitalizations and interventions with accompanying hospital infections. At the long term follow-up, amyloidosis may be the life-threatening complication particularly in patients with M694V homozygous mutations. This case was reported to point out the importance of early remembrance of possible autoinflammatory diseases even at very early ages especially at endemic countries.
